# Urodynamic and Quality-of-Life Outcomes After Endometrial Cancer Treatment: A Prospective Cohort Study

**DOI:** 10.1007/s00192-025-06503-5

**Published:** 2026-01-06

**Authors:** Jiri Spacek, Dominik Habes, Munachiso Iheme Ndukwe, Ondrej Dvorak, Pavel Navratil, Petra Bretova, Denisa Pohankova, Igor Sirak, Milan Vosmik, Jaroslav Pacovsky, Akaninynene Eseme Ubom, Martin Stepan

**Affiliations:** 1 Department of Urology, Faculty of Medicine in Hradec Kralove, Charles University, Hradec Kralove, Czech Republic; 2 Department of Urology, University Hospital Hradec Kralove, Hradec Kralove, Czech Republic; 3 Department of Obstetrics and Gynecology, Faculty of Medicine in Hradec Kralove, Charles University, Hradec Kralove, Czech Republic; 4 Department of Obstetrics and Gynecology, University Hospital Hradec Kralove, Hradec Kralove, Czech Republic; 5 Department of Oncology and Radiotherapy, Faculty of Medicine in Hradec Kralove, University Hospital Hradec Kralove, Charles University, Hradec Kralove, Czech Republic; 6 Department of Obstetrics and Gynaecology, Faculty of Clinical Sciences, PAMO University of Medical Sciences, Port Harcourt, Nigeria

**Keywords:** Bladder function, Endometrial cancer, Pelvic oncology, Quality of life, Radiotherapy, Urodynamics

## Abstract

**Introduction and Hypothesis:**

Endometrial cancer therapy may affect lower urinary tract function and quality of life (QoL). We prospectively evaluated short-term urodynamic and QoL outcomes after surgical treatment with or without adjuvant radiotherapy.

**Methods:**

Women with biopsy-confirmed endometrial cancer underwent standardized urodynamic testing before surgery and 6 months after treatment completion. Parameters included detrusor pressure, bladder capacity, compliance, and post-void residual (PVR) urine volume. QoL was assessed using EORTC QLQ-C30 and QLQ-EN24 questionnaires. Pre- and post-treatment data were compared using nonparametric tests (α = 0.05). Surgery was performed either robotically or via the open abdominal approach.

**Results:**

Of the 101 participants, 55 received adjuvant radiotherapy and 46 were treated with surgery alone. No clinically significant  urodynamic deterioration was observed post-surgery. Median PVR urine volume decreased from 50 to 40 mL (*p* = 0.002) and detrusor pressure from 30 to 28 cmH_2_O (*p* = 0.08) following surgery; other parameters remained stable. No acute radiation cystitis occurred. QoL scores were high at baseline and follow-up, with no domain showing a ≥ 10-point change. Sexual and urinary symptom scores remained unchanged.

**Conclusions:**

Within 6 months after endometrial cancer treatment, urodynamic parameters and QoL remained stable. These results suggest short-term functional safety of contemporary surgical and radiotherapy protocols, warranting longer-term follow-up.

**Supplementary Information:**

The online version contains supplementary material available at 10.1007/s00192-025-06503-5.

## Introduction

Endometrial cancer is the most common gynecological malignancy in developed countries, with a steadily increasing incidence, particularly in aging and obese populations [1, 2]. In the Czech Republic, the burden of endometrial cancer remains especially high, representing a significant public health concern. With an incidence rate of 34.86/100,000 females, endometrial cancer remains the most common gynecological cancer in the Czech Republic, with 1,870 new cases and 294 related mortality recorded annually [3, 4]. Treatment typically involves hysterectomy with bilateral salpingo-oophorectomy, often performed via minimally invasive approaches, along with lymph-node assessment for staging purposes, and may be followed by radiotherapy in patients with intermediate or high-risk features [5, 6].

Given the anatomical proximity of the uterus to the lower urinary tract and ureters, both surgical and radiotherapeutic interventions carry a risk of urological complications [7]. These may include both acute and chronic forms of urotoxicity, manifesting as lower urinary tract dysfunction, ureteral injury, or reduced bladder compliance [7]. Despite these risks, urological outcomes in patients treated for endometrial cancer have been less thoroughly studied compared to other gynecological malignancies, such as cervical cancer.

We prospectively evaluated short-term changes in lower urinary tract function and quality of life (QoL) after endometrial cancer treatment. Previous research addressing this topic was limited to a group of patients treated with radiotherapy, in whom significant reductions in maximum vesical pressure, maximum detrusor pressure, and bladder capacity were observed [8].

In the present study, we hypothesized that bladder function in predominantly surgically treated patients would remain stable over 6 months, with no clinically significant decline in urodynamic parameters, indicating that the surgical approach may be urodynamically safer than radiotherapy. The primary objective was to assess changes in post-void residual (PVR) urine volume, maximal vesical and detrusor pressures, and bladder capacity. The secondary objective was to evaluate changes in QoL outcomes using the QLQ-C30 and EN-24 questionnaires.

## Materials and Methods

### Study Design

This prospective, monocentric cohort study was conducted to evaluate urodynamic changes in patients undergoing oncological treatment for biopsy-confirmed endometrial cancer.

### Inclusion and Exclusion Criteria

Eligible participants were women aged ≥ 18 years with a clinical indication for surgical treatment of endometrial cancer, who provided written informed consent for study participation. All participants were required to undergo a baseline urodynamic examination within 1 week before surgery and a follow-up examination 6 months postoperatively (180 days ± 14 days), or 6 months after completion of any adjuvant oncological therapy.

Exclusion criteria included suspected preoperative disease spread beyond the uterus, adnexa, or lymphatic system; refusal to participate in the study; a history of perioperative injury to the urinary tract; prior urogynecological surgery for incontinence; withdrawal of consent for study participation; and failure to complete both urodynamic examinations within the specified timeframes.

### Study Population

Sample size was calculated using PASS 2023 Power Analysis and Sample Size Software (NCSS, LLC, Kaysville, Utah, USA). A total of 100 participants was deemed sufficient to detect clinically significant differences in urodynamic parameters with adequate statistical power.

Between February 24, 2017, and January 20, 2023, 154 eligible patients were identified. Of these, 127 consented to participate in the study, and 115 met the inclusion criteria. Fourteen participants were excluded due to incomplete or poor-quality follow-up urodynamic examinations. Thus, 101 patients were included in the urodynamic analysis. Of these, 21 did not return the baseline QoL questionnaire and were therefore excluded from the QoL assessment, resulting in 80 patients in the QoL cohort. On descriptive review, the excluded patients did not appear to differ in key demographic or clinical characteristics. The final study cohort consisted of 101 patients, including 55 who received surgery followed by adjuvant therapy, and 46 who underwent surgery alone.

### Urodynamic Examination Protocol

Urodynamic examinations were conducted at the Urogynecological Unit of a University Teaching Hospital, using the UMS Dynamic Mini System (Medetron s.r.o.). All examinations were performed by a national board-certified urogynecologist.

The initial examination was conducted within 1 week before surgery, typically on the day before surgery. The follow-up examination occurred 180 ± 14 days after surgery or after completion of adjuvant therapy. Acute radiation cystitis was defined as new urinary symptoms assessed at this standardized 6-month post-treatment follow-up. This interval was chosen to allow for tissue recovery and stabilization of urodynamic parameters after surgery or radiation. A similar 6-month timeframe was used in a prospective study by Emirdar et al. [8], which demonstrated the feasibility of detecting short-term bladder function changes within this period.

Urodynamic evaluation followed a standardized three-step protocol:

### Filling Cystometry

Filling cystometry was performed using physiological saline at room temperature, instilled at a rate of 50 mL/min. Bladder emptying was confirmed in all patients before the procedure, by asking each patient to void just before the examination. If any PVR urine was present, catheterization was performed to ensure complete emptying. Parameters assessed included first sensation of bladder filling, cystometric capacity, detrusor pressure at maximum flow, bladder compliance, and the presence of detrusor overactivity or stress incontinence during filling.

### Urethral Pressure Profilometry

Profilometry involved gradual catheter withdrawal with continuous pressure recording to evaluate urethral closure function. Key parameters included functional urethral length and maximum urethral closure pressure.

### Uroflowmetry

Uroflowmetry was performed by having the patient void into a uroflowmeter-equipped toilet. Parameters assessed included voided volume, voiding time, maximum and average flow rates, and patterns suggestive of obstructed or interrupted flow; detrusor overactivity was assessed during cystometry. Post-void residual urine volume was determined on the basis of instilled fluid volume.

### Quality of Life Assessment

Eighty patients were included in the QoL assessment, of whom 42 underwent surgery plus adjuvant radiotherapy and 38 were managed with surgery only. To assess QoL in the patients treated for endometrial cancer, we used the Czech versions of the EORTC QLQ-C30 (version 3.0) and the disease-specific endometrial cancer module EORTC QLQ-EN24 (questionnaires attached as appendices). The questionnaires were self-administered: patients took them home, completed them in their own time, and returned them by mail. Assistance from clinic staff was available if needed.

The EORTC QLQ-C30 assesses general QoL in cancer patients and contains 30 questions divided into three main sections: global health status, functional scales, and symptom scales. The five functional scales evaluate physical, role, emotional, cognitive, and social functioning. The symptom scales include fatigue, nausea and vomiting, pain, dyspnea, insomnia, appetite loss, constipation, diarrhea, and financial difficulties. The EORTC QLQ-EN24 is a supplementary module specific to endometrial cancer and complements the QLQ-C30. It also includes questions grouped into functional and symptom subscales. The symptom scales include the endometrial cancer specific symptoms of lymphedema, urologic, gastrointestinal, vaginal/sexual, musculoskeletal (back and pelvic pain, muscle pain), and neurologic (numbness/tingling) symptoms, poor body image, hair loss and taste changes. The functional scales include sexual interest (libido), sexual activity, and sexual enjoyment. The questions in both questionnaires are rated on a 1–4 or 1–7 scale. In symptom scales, a higher score indicates greater symptom severity, whereas in functional scales, a higher score indicates better function. The evaluation of results was conducted according to the official EORTC scoring manual. Permission for clinical and noncommercial use of the questionnaires was obtained from the European Organisation for Research and Treatment of Cancer (EORTC).

### Statistical Analysis

Normality of the data distribution was assessed using the Shapiro–Wilk test, which revealed non-normal distributions across multiple parameters. Accordingly, nonparametric statistical tests were applied. Baseline characteristics were compared using the Mann–Whitney U test for continuous variables and the χ^2^ test or Fisher’s exact test for categorical variables. The Wilcoxon signed-rank test was used for paired pre- and post-treatment comparisons, and the Mann–Whitney U test was used for comparisons between independent groups. Analyses were performed using NCSS 2025 (v25.0.1, NCSS, LLC, Kaysville, Utah, USA). Post hoc power analysis using G*Power indicated a statistical power of 0.74 for detecting medium effect sizes (*r* = 0.5) at α = 0.05 with the given sample size.

## Results

Baseline data for the evaluated cohort are summarized in Table 1, grouped by adjuvant treatment status. The surgery only group included 46 patients, with a median age of 64 years (IQR 55.5–70), while the surgery plus adjuvant radiotherapy group included 55 patients with a slightly higher median age of 65 years (IQR 60–72). The median body mass index (BMI) was higher in the surgery only group at 33.5 kg/m^2^ (IQR 28.4–37.75) compared to 29.4 kg/m^2^ (IQR 26.6–35.5) in the surgery plus adjuvant radiotherapy group.

**Table 1:** Baseline characteristics of the study participants

Characteristic	Surgery without adjuvant RT (*n* = 46)	Surgery with adjuvant RT (*n* = 55)
**Age (years)**		
Median	64	65
IQR (Q1–Q3)	55.5–70	60–72
**BMI** (kg/m^2^)		
Median	33.5	29.4
IQR (Q1–Q3)	28.4–37.75	26.6–35.5
**Lymph-node assessment**		
Sentinel lymph-node mapping	15 (32.6%)	14 (25.5%)
Full lymphadenectomy	6 (13.0%)	28 (50.9%)
No lymph-node assessment	25 (54.4%)	13 (23.6%)
**FIGO stage (2009)**		
Stage IA	42 (91.3%)	9 (16.4%)
Stage IB	4 (8.7%)	23 (41.8%)
Stage II	0 (0%)	17 (30.9%)
Stage III	0 (0%)	6 (10.9%)
**Type of surgery**		
Robotic-assisted	33 (72%)	20 (36%)
Open surgery	13 (28%)	35 (64%)
**Adjuvant radiotherapy type**	—	
VBT only	—	8 (14.5%)
EBRT ± VBT	—	47 (85.5%)

Regarding surgical approach, robotic-assisted surgery was more frequently employed in patients who did not receive adjuvant radiotherapy (33 cases; 32.67%), whereas open surgery predominated in the surgery plus adjuvant radiotherapy group (35 cases; 34.66%). Only 13 patients (12.87%) in the surgery only group underwent open surgery. These distributions may reflect clinical preferences or patient factors influencing the selection of surgical modality. At our institution, patients with preoperative high-risk features based on molecular classification are more likely to undergo open surgery. Specific individual reasons such as uterine size or prior laparotomies were not systematically recorded.

### Changes in Urodynamic Parameters Following Oncologic Treatment

To evaluate changes in urodynamic parameters before and after oncologic treatment (surgery with or without adjuvant radiotherapy), the Wilcoxon signed-rank test was applied. Among the 101 patients included in this analysis, most urodynamic parameters did not exhibit statistically significant differences (see Table 2).

**Table 2:** Pre- and post-treatment urodynamic parameters for all patients

Parameter	Pre-treatment (*n* = 101) median (IQR)	Post-treatment (*n* = 101) median (IQR)	*p* value
First sensation to void (mL)	150 (100–200)	150 (100–200)	1.000
Bladder capacity (mL)	400 (350–450)	400 (350–450)	1.000
Detrusor pressure at maximum flow (cmH_2_O)	30 (25–35)	28 (24–32)	0.080
Maximum urethral closure pressure (cmH_2_O)	70 (60–80)	70 (60–80)	1.000
Voided volume (mL)	350 (300–400)	350 (300–400)	1.000
Voiding time (s)	20 (15–25)	20 (15–25)	1.000
Maximum flow rate (mL/s)	15 (12–18)	15 (12–18)	1.000
Post-void residual urine (mL)	50 (40–60)	40 (30–50)	0.002

A statistically significant but clinically unimportant decrease was observed in PVR urine volume following treatment (50 mL vs. 40 mL, *p* = 0.002). Detrusor pressure showed a trend towards reduction (*p* = 0.080), which may reflect a treatment-related effect, although it did not reach statistical significance. Other parameters—including first sensation to void, bladder capacity, bladder compliance, maximum detrusor pressure, maximum urethral closure pressure, voided volume, voiding time, and peak flow rate—remained within normal limits in all patients, with no statistically significant pre- to post-treatment changes. No new cases of detrusor overactivity were detected, and no episodes of acute radiation cystitis requiring intervention were reported before the 6-month follow-up.

### Urodynamic Changes Following Surgery only Treatment

In the group of patients who underwent surgery without adjuvant radiotherapy (*n* = 46), Wilcoxon signed-rank tests were used to compare pre- and post-treatment urodynamic parameters. No statistically significant differences were found for any of the measured variables, including detrusor pressure at maximum flow (*p* = 0.345), first sensation to void, bladder capacity, maximum urethral closure pressure, voided volume, voiding time, peak flow rate, and post-void residual volume (all *p* = 1.000) (Supplement).

### Urodynamic Changes Following Surgery with Adjuvant Radiotherapy

In the group of patients who received surgery followed by adjuvant radiotherapy (*n* = 55), Wilcoxon signed-rank tests revealed statistically significant changes in two urodynamic parameters: Detrusor pressure at maximum flow decreased (30 cmH_2_O vs. 28 cmH_2_O, *p* = 0.020), as did PVR urine volume (50 mL vs. 40 mL, *p* = 0.009). No statistically significant changes were observed for first sensation to void (*p* = 1.000), bladder capacity (*p* = 0.178), maximum urethral closure pressure (*p* = 1.000), voided volume (*p* = 0.206), voiding time (*p* = 1.000), or peak flow rate (*p* = 1.000) (Supplement).

### Comparison of Urodynamic Changes Between Surgery only Treatment and Surgery with Adjuvant Radiotherapy

A comparative analysis was performed between patients treated with surgery only (*n* = 46) and those treated with surgery followed by adjuvant radiotherapy (*n* = 55). The Mann–Whitney U test was used to assess differences in the median change (Δ) values of urodynamic parameters.

No statistically significant differences were found between the groups for any variable, including first sensation to void (*p* = 0.930), bladder capacity (*p* = 0.488), detrusor pressure at maximum flow (*p* = 0.210), maximum urethral closure pressure (*p* = 0.792), voided volume (*p* = 0.465), voiding time (*p* = 0.791), maximum flow rate (*p* = 0.791), or PVR urine volume (*p* = 0.365) (Supplement).

### Quality of Life of Patients Treated for Endometrial Cancer

Using the EORTC QLQ-C30 questionnaire, the mean scores for global health status at baseline was similar between the surgery-only and surgery plus adjuvant radiotherapy groups (65.8 vs. 67.9; *p* = 0.47) (Supplement). At 6-month follow-up, global health scores remained comparable for both groups (65.8 vs. 65.1; *p* = 0.72), with no significant within- or between-group changes (Supplement). Across functional domains, scores were high at both baseline and at 6 months follow-up after treatment (> 80 in all subdomains) for both groups. No significant increase or decrease was noted in any functional domain between baseline and 6 months follow-up in both groups (all* p* > 0.05) (Supplement). Symptom scores were low for both groups at both baseline and at 6 months follow-up. Fatigue was the most commonly reported symptom in both groups, with mean scores increasing modestly from 22.5 at baseline to 24.0 at 6 months post-treatment in the surgery-only group, and from 22.8 to 23.8 in the surgery plus adjuvant radiotherapy group (*p* = 0.68). No symptom domain showed a significant or clinically relevant change (defined as a ≥ 10-point difference) between baseline and 6 months post-treatment (Supplement).

Using the EORTC QLQ-EN24 questionnaire, at baseline, endometrial cancer–specific symptoms were mild in both groups (Supplement). No symptom domain showed a ≥ 10-point change between baseline and 6 months post-treatment. Sexual interest (libido) and sexual activity scores were high in both groups at baseline and at 6 months post-treatment (88.9–97.6 points), while sexual enjoyment scores were lower (55.6–72.2 points) (Supplement). All sexual function domains remained stable between baseline and at 6 months post-treatment. While sexual enjoyment scores increased between baseline and 6 months post-treatment for both groups, sexual interest (libido) and sexual activity scores reduced for the surgery only group and increased for the surgery plus adjuvant radiotherapy group (Supplement). These changes were, however, not statistically significant (*p* > 0.05).

## Discussion

To our knowledge, this is the first study to evaluate lower urinary tract function in endometrial cancer patients using serial urodynamic testing, as well as QoL following treatment. This prospective urodynamic study assessed the impact of modern surgical and radiotherapy treatments for endometrial cancer on lower urinary tract function. By comparing standardized pre- and post-treatment urodynamic parameters in a well-defined cohort, we demonstrated that both surgery and adjuvant radiotherapy are generally safe from a urodynamic perspective. Importantly, no clinically meaningful deterioration in both urodynamic function and QoL were observed.

Although the 10 mL reduction in PVR urine volume reached statistical significance, this magnitude is below thresholds generally considered clinically meaningful (PVR ≈ 100 mL) [9]. Several factors may explain the modest PVR urine volume reduction—such as resolution of outlet obstruction caused by the uterine mass, improved pelvic floor mobility, and normalization of urethral angulation following surgery [10, 11]—yet the small magnitude of these changes (≤ 10 mL and ≤ 2 cmH_2_O) does not warrant further speculation regarding their underlying mechanisms.

In the group of patients who had surgery followed by adjuvant radiotherapy, detrusor pressure at maximum flow and PVR urine volume improved. A reduction in detrusor pressure could indicate early changes in detrusor contractility or bladder wall compliance, possibly due to mild radiation effects [12]. However, the concurrent decrease in PVR urine volume suggests that any such changes did not impair bladder emptying. This pattern—more efficient voiding at lower pressures—could represent improved detrusor–sphincter coordination or subclinical, compensated detrusor underactivity [13].

Crucially, between-group analyses did not reveal significant differences in urodynamic change scores between patients treated with surgery alone and those receiving adjuvant radiotherapy. Thus, the modest within-group variations observed after radiotherapy were comparable to those seen following surgery alone. These findings indicate that modern image-guided adjuvant radiotherapy protocols, applied with strict bladder-sparing dose constraints, do not result in clinically relevant urodynamic impairment within the first 6 months after treatment. Our findings align with prior reports documenting low rates of urodynamic toxicity with modern, image-guided EBRT that employs bladder-sparing constraints [14–16].

When positioned within the spectrum of gynecologic surgical literature, our findings are consistent with what would be expected after simple hysterectomy. Patients in our cohort underwent surgery comparable to benign indications, and the modest postoperative changes we observed resemble those reported in long-term urodynamic studies following benign hysterectomy, where only minimal deterioration has been described [11]. These changes are substantially less pronounced than those seen after radical hysterectomy for cervical cancer, where impaired bladder compliance and storage dysfunction are common [17]. Likewise, studies including patients with more advanced gynecologic malignancies, particularly those undergoing radical procedures with adjuvant radiotherapy, report marked reductions in flow rate and increased residuals [18]. The relatively mild findings in our early-stage endometrial cancer cohort therefore align with the expected functional impact of simple hysterectomy and limited adjuvant treatment. No domain of both the EORTC QLQ-C30 and QLQ-EN24 QoL assessments demonstrated a clinically relevant decline. These findings were consistent across both treatment groups, underscoring the functional safety of modern endometrial cancer therapy. Importantly, urologic symptoms—a central focus of this study—did not increase following adjuvant radiotherapy, supporting the urodynamic evidence of preserved bladder function. Sexual function/health was also well maintained. High libido and sexual activity scores suggest a reassuring functional outcome, especially given the known negative impact of pelvic surgery and radiotherapy on sexual health. Collectively, these findings indicate that contemporary surgical and radiotherapy approaches are well tolerated both functionally and symptomatically during the first 6 months after treatment.

Although validated symptom-specific questionnaires such as OAB-q or ICIQ-SF would have captured subjective urinary complaints in greater detail, we used the EORTC QLQ-EN24, which contains four items on urinary symptoms, to minimize patient burden. Future studies should combine urodynamic and symptom-specific instruments.

### Clinical Implications of Study Findings

Our findings suggest that contemporary surgical management of endometrial cancer, with or without adjuvant radiotherapy, does not produce clinically significant deterioration in lower urinary tract function within the first 6 months after treatment. Patient-reported outcomes were stable across all domains, indicating that bladder and sexual functions are well-preserved in the short term. These results support survivorship care models that emphasize functional preservation alongside oncologic efficacy, highlighting that urinary function should be monitored as part of holistic QoL assessment in endometrial cancer survivors.

Routine urodynamic testing is unlikely to be necessary for asymptomatic patients during early follow-up, as the observed changes were minor and clinically insignificant. However, even small physiological shifts may be relevant in vulnerable groups such as elderly women, those with pre-existing lower urinary tract symptoms, diabetes, or prior pelvic floor dysfunction. For these patients, a symptom-triggered evaluation strategy may be beneficial, where targeted urodynamic assessment is reserved for persistent or worsening voiding complaints, equivocal post-void residuals, or treatment planning for pelvic floor rehabilitation.

Early implementation of conservative measures—such as pelvic floor exercises, bladder training, and regular voiding schedules—may further support functional recovery and prevent late dysfunction. Because radiation-related effects can manifest months or years after therapy, long-term follow-up with periodic symptom review remains warranted. Overall, our data advocate for a symptom-guided, rehabilitation-oriented approach to post-treatment urinary care, integrating functional outcomes into survivorship planning for women treated for endometrial cancer.

### Strengths and Limitations of the Study

Key strengths of this study include its prospective design, uniform urodynamic testing before and after treatment, and inclusion of both surgical and radiotherapy groups. In contrast to previous literature that primarily reports subjective symptoms, this study offers objective data on bladder function in endometrial cancer survivors. Ours is one of the few studies to evaluate both objective urodynamic parameters and patient-reported QoL following gynecological oncology treatment. These findings contribute novel and clinically relevant evidence to an understudied area in urogynecologic oncology and support the continued evolution of function-preserving treatment strategies. That said, this study also has some limitations. The 6-month follow-up period may be insufficient to capture late-onset urological toxicity, which is known to occur months to years after pelvic radiotherapy [19]. Additionally, we did not document the precise number of days between completion of radiotherapy and follow-up urodynamic testing, and minor timing variation may introduce small measurement bias. The absence of a non-cancer control group limits interpretation of absolute changes. Our design focused on within-subject functional stability rather than comparison with healthy populations. Finally, although all testing followed standardized protocols, some degree of procedural or measurement variability is unavoidable.

## Conclusion

Within the first 6 months following treatment for apparent early stage endometrial cancer, no clinically meaningful deterioration in bladder function or QoL was observed. These findings suggest short-term functional stability of modern surgical and radiotherapy protocols. However, interpretation is limited by the study’s modest sample size, particularly for the QoL cohort, and by the short duration of follow-up. Larger studies with longer follow-up that include symptom-specific patient-reported outcomes are needed to confirm long-term safety and to better characterize postoperative bladder recovery.

## Supplementary Information

Below is the link to the electronic supplementary material.

Supplementary file1 (DOCX 25 KB)

## Data Availability

The data that support the findings of this study are available from the corresponding author upon reasonable request.
