# Supplementary material for: Urodynamic and Quality-of-Life Outcomes After Endometrial Cancer Treatment: A Prospective Cohort Study
Source: Int Urogynecol J. 2026 Jan 6;37(7):2065–71. doi: 10.1007/s00192-025-06503-5 (PMC13385002; doi:10.1007/s00192-025-06503-5)
Supplement: Supplementary file 1 — Supplementary file1 (DOCX 25 KB) [file 192_2025_6503_MOESM1_ESM.docx]

**Supplemental Table 1: Surgery only group: pre- and post-treatment urodynamic parameters**

| Parameter | Pre-treatment (*n*=46)  Median (IQR) | Post-treatment (*n*=46)  Median (IQR) | *p-*value |
| --- | --- | --- | --- |
| **First sensation to void (ml)** | 150 (100-200) | 150 (100-200) | 1.000 |
| **Bladder capacity (ml)** | 400 (350-450) | 400 (350-450) | 1.000 |
| **Detrusor pressure at maximum flow (cmH2O)** | 30 (25-35) | 30 (24-32) | 0.345 |
| **Maximum urethral closure pressure (cmH2O)** | 70 (60-80) | 70 (60-80) | 1.000 |
| **Voided volume (ml)** | 350 (300-400) | 350 (300-400) | 1.000 |
| **Voiding time (s)** | 20 (15-25) | 20 (15-25) | 1.000 |
| **Maximum flow rate (ml/s)** | 15 (12-18) | 15 (12-18) | 1.000 |
| **Post-void residual urine (ml)** | 50 (40-60) | 50 (40-60) | 1.000 |

**Supplemental Table 2: Surgery + adjuvant radiotherapy group: pre- and post-treatment urodynamic parameters**

| Parameter | Pre-treatment (*n*=55)  Median (IQR) | Post-treatment (*n*=55)  Median (IQR) | *p-*value |
| --- | --- | --- | --- |
| **First sensation to void (ml)** | 150 (100-200) | 150 (100-200) | 1.000 |
| **Bladder capacity (ml)** | 400 (350-450) | 400 (350-450) | 0.178 |
| **Detrusor pressure at maximum flow (cmH2O)** | 30 (25-35) | 28 (22-32) | 0.020 |
| **Maximum urethral closure pressure (cmH2O)** | 70 (60-80) | 70 (60-80) | 1.000 |
| **Voided volume (ml)** | 350 (300-400) | 350 (320-400) | 0.206 |
| **Voiding time (s)** | 20 (15-25) | 20 (15-25) | 1.000 |
| **Maximum flow rate (ml/s)** | 15 (12-18) | 15 (12-18) | 1.000 |
| **Post-void residual urine (ml)** | 50 (40-60) | 40 (30-50) | 0.009 |

**Supplemental Table 3: Between-group comparison of change in urodynamic parameters (surgery only vs. surgery + adjuvant radiotherapy)**

| Parameter | Median Δ (Surgery only) | Median Δ (Surgery + RT) | *p*-value |
| --- | --- | --- | --- |
| **First sensation to void (ml)** | 0 | 0 | 0.930 |
| **Bladder capacity (ml)** | 0 | 0 | 0.488 |
| **Detrusor pressure at max flow (cmH₂O)** | 0 | –2 | 0.210 |
| **Max urethral closure pressure (cmH₂O)** | 0 | 0 | 0.792 |
| **Voided volume (ml)** | 0 | 0 | 0.465 |
| **Voiding time (s)** | 0 | 0 | 0.791 |
| **Maximum flow rate (ml/s)** | 0 | 0 | 0.791 |
| **Post-void residual volume (ml)** | 0 | –10 | 0.365 |

**Supplemental Table 4: Between-group comparison of change in urodynamic parameters (Brachytherapy vs. EBRT + brachytherapy)**

| Parameter | Median Δ (BT only) | Median Δ (EBRT + BT) | *p*-value |
| --- | --- | --- | --- |
| **First sensation to void (ml)** | 0 | 0 | 0.874 |
| **Bladder capacity (ml)** | 0 | 0 | 0.849 |
| **Detrusor pressure at max flow (cmH₂O)** | –1 | –2 | 0.543 |
| **Max urethral closure pressure (cmH₂O)** | 0 | 0 | 0.824 |
| **Voided volume (ml)** | 0 | 0 | 0.700 |
| **Voiding time (s)** | 0 | 0 | 0.721 |
| **Maximum flow rate (ml/s)** | 0 | 0 | 0.721 |
| **Post-void residual volume (ml)** | –10 | –10 | 0.721 |

**Supplemental Table 5: Qaulity of life scores using EORTC QLQ-C30 for both study groups**

|  | Surgery only, *n=*38 | | Surgery + adjuvant radiotherapy, *n*=42 | |
| --- | --- | --- | --- | --- |
|  | Baseline  Mean ± SD | 6 months  Mean ± SD | Baseline  Mean ± SD | 6 months  Mean ± SD |
| **Global health status** | 65.8 ± 16.9 | 65.8 ± 17.5 | 67.9 ± 13.7 | 65.1 ± 14.0 |
| **Symptom scales** |  |  |  |  |
| Fatigue | 22.5 ± 14.5 | 24.0 ± 16.2 | 22.8 ± 12.7 | 23.8 ± 11.1 |
| Nausea and vomiting | 14.5 ± 22.0 | 12.7 ± 22.1 | 7.5 ± 17.7 | 9.9 ± 17.3 |
| Pain | 9.2 ± 18.5 | 7.9 ± 12.1 | 4.0 ± 8.1 | 4.8 ± 8.5 |
| Dyspnea | 7.9 ± 14.4 | 7.0 ± 13.8 | 4.8 ± 11.8 | 4.8 ± 11.8 |
| Insomnia | 17.5 ± 16.9 | 18.4 ± 16.8 | 15.1 ± 16.8 | 16.7 ± 16.9 |
| Loss of appetite | 14.0 ± 21.4 | 13.2 ± 21.3 | 13.5 ± 19.6 | 15.9 ± 18.4 |
| Constipation | 7.0 ± 13.8 | 6.1 ± 13.1 | 3.2 ± 9.9 | 3.2 ± 9.9 |
| Diarrhea | 9.6 ± 18.8 | 9.6 ± 18.8 | 6.3 ± 15.2 | 7.1 ± 15.7 |
| Financial difficulties | 0.9 ± 5.4 | 0.9 ± 5.4 | 0 ± 0 | 0.8 ± 5.1 |
| **Functioning scales^*^** |  |  |  |  |
| Physical functioning | 90.4 ± 14.0 | 90.7 ± 12.6 | 92.1 ± 11.2 | 91.9 ± 10.7 |
| Role functioning | 80.7 ± 19.2 | 81.1 ± 18.2 | 82.9 ± 16.3 | 84.1 ± 15.2 |
| Emotional functioning | 92.3 ± 8.1 | 93.4 ± 7.0 | 89.3 ± 7.9 | 88.5 ± 8.4 |
| Cognitive functioning | 94.3 ± 8.0 | 93.9 ± 8.1 | 93.3 ± 8.3 | 94.0 ± 8.1 |
| Social functioning | 86.8 ± 21.6 | 87.3 ± 19.5 | 88.1 ± 17.8 | 89.7 ± 17.2 |

*Functioning in daily life

**Supplemental Table 6: Quality of life scores using EORTC QLQ-EN24 for both study groups**

|  | Surgery only, *n=*38 | | Surgery + adjuvant radiotherapy, *n*=42 | |
| --- | --- | --- | --- | --- |
|  | Baseline  Mean ± SD | 6 months  Mean ± SD | Baseline  Mean ± SD | 6 months  Mean ± SD |
| **Symptom scales** |  |  |  |  |
| Lymphedema | 7.0 ± 10.7 | 7.0 ± 10.7 | 4.4 ± 9.1 | 5.2 ± 10.1 |
| Urologic symptoms | 11.2 ± 16.7 | 9.4 ± 14.0 | 6.7 ± 10.9 | 8.7 ± 13.4 |
| Gastrointestinal symptoms | 10.7 ± 13.0 | 10.0 ± 13.8 | 5.2 ± 10.3 | 6.7 ± 11.2 |
| Poor body image | 13.4 ± 15.8 | 13.3 ± 16.1 | 15.0 ± 18.2 | 13.9 ± 17.6 |
| Vaginal/sexual problems^*^ | _ | _ | _ | _ |
| Back and pelvic pain | 19.3 ± 25.3 | 15.8 ± 16.9 | 15.9 ± 16.9 | 14.3 ± 16.7 |
| Numbness/tingling | 5.3 ± 18.2 | 3.5 ± 15.1 | 1.6 ± 7.2 | 3.2 ± 12.3 |
| Muscle pain | 7.0 ± 17.6 | 6.1 ± 15.2 | 3.2 ± 9.9 | 4.8 ± 13.9 |
| Hair loss | 6.1 ± 13.1 | 5.3 ± 12.3 | 3.2 ± 9.9 | 4.0 ± 10.9 |
| Taste changes | 18.4 ± 25.3 | 15.8 ± 24.2 | 7.9 ± 19.2 | 10.3 ± 21.4 |
| **Functioning scales** |  |  |  |  |
| Sexual interest (libido) | 93.3 ± 19.5 | 91.2 ± 20.6 | 95.8 ± 11.2 | 97.6 ± 8.8 |
| Sexual activity | 89.2 ± 21.3 | 88.9 ± 18.0 | 93.3 ± 13.5 | 94.3 + 12.7 |
| Sexual enjoyment | 60.0 ± 26.3 | 72.2 ± 23.9 | 55.6 ± 16.7 | 66.7 ± 21.1 |

*Sexual/vaginal questions had low response rates, limiting interpretability
